# Geographic and Climatic Effects on Fermentation Quality and Bacterial Diversity of *Saccharum arundinaceum* and *Leucaena leucocephala* Silage

**DOI:** 10.3390/microorganisms14040899

**Published:** 2026-04-16

**Authors:** Shuo Wu, Yue Liu, Dandan Chen, Mao Li, Xuejuan Zi

**Affiliations:** 1 Key Laboratory of Ministry of Education for Genetics and Germplasm Innovation of Tropical Special Trees and Ornamental Plants, Key Laboratory of Germplasm Resources of Tropical Special Ornamental Plants of Hainan Province, School of Tropical Agriculture and Forestry, Hainan University, Danzhou 571737, China; 2 Tropical Crops Genetic Resources Institute, Chinese Academy of Tropical Agricultural Sciences, Danzhou 571737, China; 3 College of Animal Science and Technology, China Agricultural University, Beijing 100091, China; 4 College of Forestry and Landscape Architecture, South China Agricultural University, Guangzhou 510642, China

**Keywords:** latitude, silage, bacterial community, fermentation parameters

## Abstract

Silage serves as a cornerstone for the advancement of the livestock industry and a critical method for biomass preservation and utilization. This study investigated the impact of geographical and environmental factors—including longitude, latitude, temperature, relative humidity, precipitation, altitude, and sunshine duration—on fermentation parameters and bacterial communities in natural forage silage. Fresh samples of *Saccharum arundinaceum* and *Leucaena leucocephala* were collected from Changjiang, Haikou, Wanning, Danzhou, Qiongzhong and Sanya in Hainan Province, China. After 60 days of anaerobic fermentation, fermentation parameters and bacterial communities were analyzed. Results showed that fermentation parameters of the same plant exhibited significant variations across different regions. For instance, *Leucaena leucocephala* silage from Haikou showed the lowest pH value (4.32), while that from Danzhou recorded the highest pH value (5.63). In *Saccharum arundinaceum* silages, the prevalent genera in HGH (*Saccharum arundinaceum* silage from Haikou) were *Weissella* (49.85%) and *Leuconostoc* (20.42%), while the bacterial community of DGH (*Saccharum arundinaceum* silage from Danzhou) was dominated by *Klebsiella* (62.69%). These results revealed significant variations in fermentation characteristics and microbial community structure of the same plant species across different geographical regions. The Mantel-test network heatmap analysis demonstrated that longitude, latitude, altitude, precipitation, and relative humidity were identified as influential factors shaping silage microbial communities, with latitude being the most important geographical factor influencing silage microbiota. In conclusion, these findings highlight the critical need for region-specific adaptation of silage production strategies, particularly in response to latitudinal variations, to accommodate local environmental conditions, even when processing identical plant species.

## 1. Introduction

Ensiling, a traditional conservation method for fresh forage grass and lignocellulosic biomass worldwide [1], is an anaerobic fermentation process driven by phyllosphere microbiota residing on the surface of leaves, mainly lactic acid bacteria [2,3,4]. The fermentation quality of silage depends on the characteristics of fresh forage grass and the microorganisms attached to the surface of raw materials. For example, it is difficult for the pH value, one of the most important indicators of silage fermentation, of alfalfa silage to decline because of its high buffer capacity [5,6,7], while that of whole-plant corn silage is usually below the common threshold of 4.2 [8,9]. On the other hand, the bacterial community on the surface of plants is extremely complex and diverse, to the extent that the composition of phyllosphere microorganisms can vary at different times of the day [10,11]. Climatic factors including temperature, humidity, and precipitation not only influence crop growth but also profoundly impact the colonization of phyllosphere microorganisms. Previous studies have mostly focused on the impact of lactic acid bacteria (LAB) inoculants or additives on the fermentation quality and bacterial community of silage [12,13,14], while neglecting the influence of geographical environmental factors on plant fermentation characteristics. Consequently, the application of identical inoculants for fermenting the same plant material across different geographical regions resulted in suboptimal fermentation performance.

In recent years, researchers have increasingly focused on the impact of geographical environmental factors on the fermentation quality and bacterial community of silage. Geographical environmental factors not only affect plant growth and development as well as the composition of phyllosphere microorganisms but also have a significant impact on plant anaerobic fermentation. Li et al. [15], who studied the differences in the fermentation quality of *Elymus nutans* silage at various altitudes in Qinghai–Tibetan Plateau, found that high altitudes could promote LAB to become the dominant microorganisms during silage fermentation, increasing the abundance of CAZyme genes related to organic acid synthesis. Temperature affects the synthesis of lignocellulose, starch, and other chemical constituents in crops, thereby influencing their fermentation quality [16]. Additionally, elevated temperatures promote the shift from homolactic to heterolactic fermentation during silage fermentation, leading to higher acetic acid levels in silage while concurrently accelerating the rapid pH decline during the initial ensiling stage [17,18]. Studies have demonstrated that geographical environmental factors including humidity, precipitation, longitude, latitude, and ultraviolet radiation intensity collectively influence the bacterial community dynamics in forage silage [19,20,21,22]. More noteworthy is that the same plant species from different geographical regions develop distinct lactic acid bacterial communities during silage fermentation [7], while the dominant LAB in silage exhibits higher similarity in climatically similar regions [23]. Crops require differentiated processing methods across varying climate zones, even when using materials of identical quality. Therefore, studying the impact of geographical environmental factors on the natural silage fermentation of plants is of great significance for improving the silage quality of the same plant species in different regions. However, to the best of our knowledge, few studies have comprehensively considered the effects of various geographical environmental factors on plant silage fermentation.

Hainan Island is located in the southernmost part of China, and due to its topography (such as the Niuling Mountains) and monsoon climate, there are significant differences in environmental factors such as temperature, rainfall, and humidity across various regions of the Island. There are five major climatic regions in Hainan: the eastern humid region, the western semi-arid region, the southern semi-arid and semi-humid region, the northern semi-humid region, and the central mountainous humid region. Furthermore, with agriculture dominating the economy of the Island and limited industrial presence [24], it serves as an ideal location for investigating the impact of geographical environmental factors on silage microorganisms. *Saccharum arundinaceum* and *Leucaena leucocephala* are two common forage plants in tropical and subtropical regions [25,26,27]. *Saccharum arundinaceum*, a caespitose perennial herbaceous grass, is commonly found in natural grasslands of tropical regions [27]. *Leucaena leucocephala*, a leguminous plant, is characterized by rapid growth, strong adaptability, and high nutritional value. It is reported that the crude protein content of its tender leaves and branches can reach 20–30% [28], significantly higher than that of most grass forages (e.g., corn stover, which contains only about 5–8% crude protein). Collectively, *Saccharum arundinaceum* and *Leucaena leucocephala* represent two contrasting yet complementary forage resources in tropical regions, characterized by differences in nutrient composition and fermentation behavior, making them ideal model substrates for investigating the effects of geographic and climatic factors on silage fermentation quality and microbial communities.

To the best of our knowledge, studies on the influence of geographical and climatic factors on silage fermentation microbiota are limited, and none have addressed the geographic variation in bacterial communities of *Saccharum arundinaceum* and *Leucaena leucocephala* silage in tropical climates. Thus, the objectives of this study were to employ PacBio full-length 16S rRNA gene sequencing to investigate the ensiling fermentation profile and bacterial community succession of natural forage grass across different regions of Hainan Island, and to analyze the relationship between silage microorganisms and geographical environmental factors.

## 2. Materials and Methods

### 2.1. Fresh Forage Grass Collection and Ensiling Process

**Figure 1:**
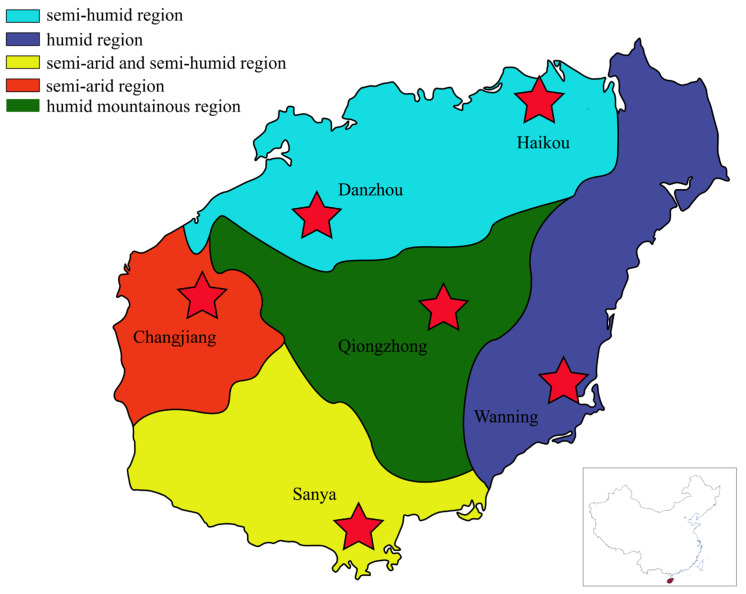
Schematic of natural forage grass samples harvesting.

In June 2021, the fresh *Saccharum arundinaceum* (GH) and *Leucaena leucocephala* (SH), were manually collected from Changjiang, Haikou, Wanning, Danzhou, Qiongzhong and Sanya (Figure 1, Table S1). These collected plants were subsequently chopped to about 1~2 cm lengths, respectively. Then the chopped plants (200 g) were ensiled in polyethylene plastic bags (35 cm × 12 cm), respectively. Three replicates per grass species were set up at each sampling site. A total of 36 silage samples (2 plant species × 6 locations × 3 replicates) were prepared and stored at room temperature (28 ± 2 °C) for 60 days. The GH and SH silages from Changjiang, Haikou, Wanning, Danzhou, Qiongzhong, and Sanya were abbreviated as CGH, HGH, WGH, DGH, QGH, SGH and CSH, HSH, WSH, DSH, QSH, SSH, respectively. Climate data were obtained from the Hainan Meteorological Service (http://hi.cma.gov.cn/).

### 2.2. Fermentation Profile Analyses

After ensiling, 50 g of silage was mixed with 200 mL of distilled water and refrigerated at 4 °C for 24 h. The mixture was then filtered through four layers of gauze. The pH value of the filtrate was measured using a pH meter. Half of the filtrate from each sample was stored in an ultra-low temperature freezer for bacterial diversity analysis. The remaining filtrate was analyzed for organic acids including lactic acid, acetic acid, propionic acid, and butyric acid using high-performance liquid chromatography (HPLC, SHIMADZU-10A, Kyoto, Japan), in accordance with the condition and procedure of Wang et al. [29]. Dry matter (DM), crude protein (CP), acid detergent fiber (ADF), and neutral detergent fiber (NDF) in silage samples were determined according to the method described by He et al. [30].

### 2.3. Bacterial Community Sequencing Analysis

The fresh plants and silages were sampled, and the total bacterial DNA was extracted with TGuide S96 Magnetic Soil/Stool DNA Kit from Tiangen Biotech Co., Ltd. (Beijing, China). The DNA concentration was determined using the Qubit dsDNA HS Assay Kit and a Qubit4.0 Fluorometer (Invitrogen, Thermo Fisher Scientific, Waltham, MA, USA). The universal primer set (27F: AGRGTTTGATYNTGGCTCAG, 1492R: TASGGHTACCTTGTTASGACTT) was used to amplify the full-length 16S rRNA gene from the total genomic DNA extracted from each sample. The PCR protocols (amplification, purification, quantification) followed the work of Bai et al. [31]. The high-quality PCR products were sequenced on a PacBio Sequel platform (Pacific Biosciences, Menlo Park, CA, USA) according to the standard protocols of Biomarker Technologies (Beijing, China). The specific steps were similar to those used by Li et al. [32]. Sequences with ≥97% similarity were clustered into the same operational taxonomic unit (OTU) using USEARCH (version 10.0), and the OTUs were filtered if their redundancy was less than 0.005%. Data were analyzed using the free online BMKCloud Platform (https://www.biocloud.net/). The sequencing data were deposited in the Sequence Read Archive (accession number: PRJNA1265434).

### 2.4. Statistical Analysis

All statistical analyses were performed using SPSS 23.0, GraphPad Prism 9.5.1, and R software (version 4.3.1). For each treatment, the fermentation parameters were analyzed by one-way analysis of variance (ANOVA). The differences between the mean values were considered significant if Duncan’s multiple-range test gave *p* < 0.05. Alpha diversity indices (ACE, Chao1, Shannon, Simpson) were compared using the one-way ANOVA followed by Tukey’s honestly significant difference test. Beta diversity was visualized by principal component analysis (PCA). The Mantel test was applied to evaluate correlations between environmental factors (longitude, latitude, temperature, precipitation, altitude, relative humidity, sunshine duration) and bacterial community composition (OTU level) as well as lactic acid bacteria (LAB) abundance. Linear discriminant analysis effect size (LEfSe) was performed to identify differentially abundant bacterial species (LDA score > 4.0, *p* < 0.05). A *p*-value < 0.05 was considered statistically significant, with significance levels indicated as * *p* < 0.05, ** *p* < 0.01, *** *p* < 0.001.

## 3. Results

### 3.1. Bacterial Communities of Fresh Materials

**Figure 2:**
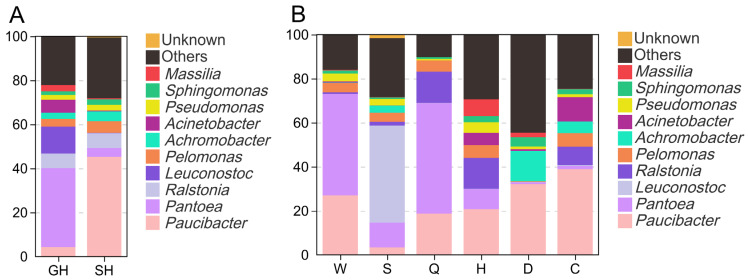
Genus (**A**) and species (**B**) level composition of bacterial communities in fresh natural forage grasses (collected from C, D, H, Q, S, W). GH, *Saccharum arundinaceum*; SH, *Leucaena leucocephala*. C, Changjiang; H, Haikou; W, Wanning; D, Danzhou; Q, Qiongzhong; S, Sanya.

As shown in Figure 2A, the dominant bacteria taxa in the phyllosphere of fresh plants differed, with *Paucibacter* most abundant in SH (45.26%) and *Pantoea* most abundant in GH (35.78%). The relative abundance of *Leuconostoc* in GH was 12.21%, while that in SH was only 0.04%. Figure 2B illustrates the bacterial diversity on the surfaces of plants from different regions of Hainan Island (genus level). *Leuconostoc* was the most dominant genus in Sanya (S), accounting for 44.07% of the relative abundance of total bacteria. The genus of *Paucibacter* was most abundant in Changjiang (C), Haikou (H), and Danzhou (D), with the relative abundance of 33.89%, 20.68%, and 32.05%, respectively. *Pantoea* was the dominant bacteria in Wanning (W, 46.07%) and Qiongzhong (Q, 50.17%).

### 3.2. Fermentation Quality of Silages

The pH values and organic acid contents of silages are shown in Table 1. After 60 d of ensiling, the pH values of the silages ranged between 4.32 and 5.84, with the lowest pH value of 4.32 in the HSH silage and the highest pH value of 5.84 in the DGH silage. Among the two natural forage silages, SH silage had the highest lactic acid content (112.17 g/kg DM). The butyric acid content of HSH silage was the highest (16.21 g/kg DM). Only trace amounts of butyric acid were detected in the DSH group (1.15 g/kg DM). Also, as shown in Table 2, silage produced from GH and SH collected from different regions in Hainan Island exhibited significant differences (*p* < 0.05) in dry matter (DM) and crude protein (CP) contents. Overall, the CP content of SH was higher than that of GH (14.23% DM vs. 9.14% DM). Among the groups, the HSH group had the highest CP content (18.95% DM), whereas the HGH group had the lowest (7.46% DM). The NDF content of GH was consistently above 70% DM. In contrast, the NDF content of SH was greatly influenced by region; for example, the WSH group had an NDF content of only 41.21% DM, while the DSH group reached 53.33% DM.

### 3.3. Characteristics of Bacterial Communities of Natural Forage Silage

**Figure 3:**
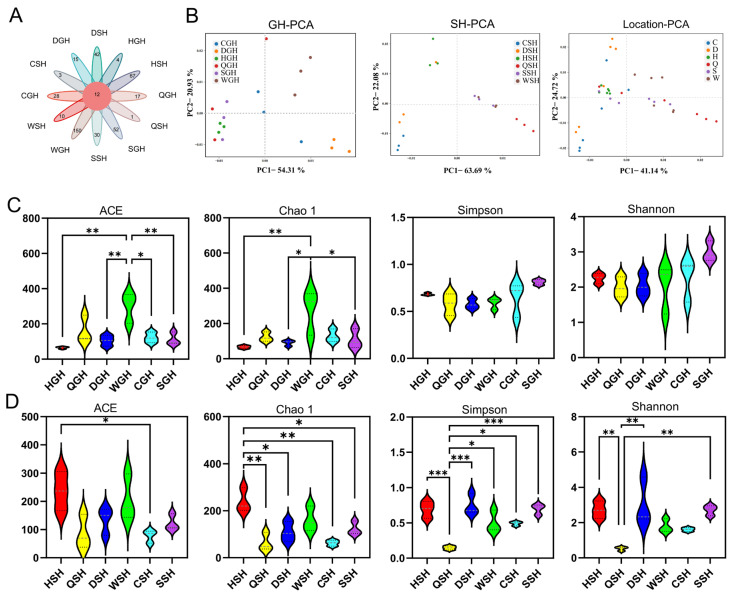
Bacterial community diversities of GH and SH silages. (**A**) Venn analysis of OTUs in the bacterial community of silages. (**B**) Principal component analysis of bacterial communities for silages. (**C**,**D**) The α-diversity analysis of GH and SH silages. *, *p* < 0.05; **, *p* < 0.01; ***, *p* < 0.001.

Sequencing of bacterial 16S rRNA gene yielded 434,379 CCS (Circular Consensus Sequencing) sequences with an average of 12,066 sequences for each silage sample. As shown in Figure 3A, the number shared OTUs among different silage samples was 12, with WGH silage having the highest number of specific OTUs at 150. QSH silage had only one specific OUT. The principal component analysis (PCA) showed a clear difference in the beta diversity of bacterial communities of silages from different regions, especially in SH silages (Figure 3B). Figure 3C,D show the alpha diversity of bacterial diversity of the two forage grass silages. Based on the comprehensive analysis of ACE, Chao 1, Simpson, and Shannon indicators, this study revealed that geographical environmental factors exerted significant impacts on the bacterial abundance in GH silage, and showed greater influence on bacterial diversity in SH silage.

**Figure 4:**
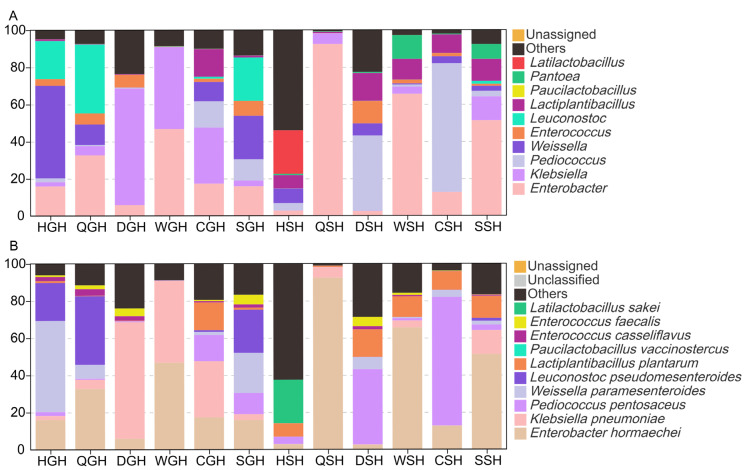
Genus-level (**A**) and species-level (**B**) composition of bacterial communities in GH and SH silages from different regions in Hainan Island.

**Figure 5:**
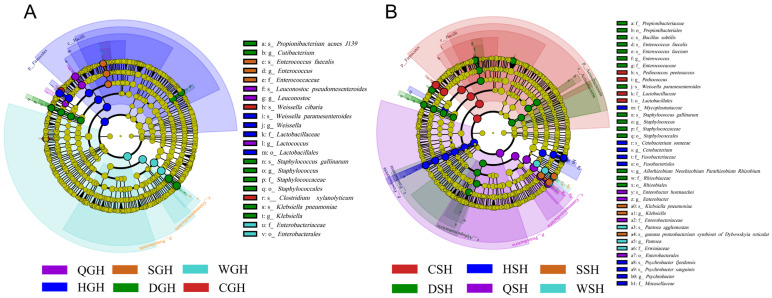
Cladogram of LEfSe analysis of GH (**A**) and SH (**B**) silages.

As illustrated in Figure 4A,B, after ensiling, *Enterobacter* and *Klebsiella* were the dominant genera in GH silages (37.59~90.81%), except HGH and SGH groups. Among the GH silages, HGH group held a high relative abundance of *Weissella* (49.85%), while QGH group held a high relative abundance of *Leuconostoc* (36.98%). In SGH silage, the abundances of *Weissella* and *Leuconostoc* were comparable, accounting for 23.56% and 23.65%, respectively. In CGH silage, the predominant LAB were *Lactiplantibacillus* (14.85%), *Pediococcus* (14.16%), and *Weissella* (10.38%). Therefore, geographical environmental factors exerted a significant impact on the structure and composition of predominant LAB in GH silage. As for SH silages, *Enterobacter* was the most dominant genus in QSH (92.33%), WSH (65.48%), and SSH (51.15%) groups. *Pediococcus* was the most important LAB in CSH (69.18%) and DSH (40.51%). In addition to that, the relative abundance of *Lactiplantibacillus* in DSH, WSH, CSH, and SSH groups was 10.03–14.93%, and the relative abundance of *Latilactobacillus* in HSH group was 23.44%. Unfortunately, the relative abundance of all LAB in the QSH group was below 1%. Similar to GH silage, distinct differences were observed in the bacterial community structure across the SH silage groups. *Enterococcus faecalis*, *Leuconostoc pseudomesenteroides*, *Weissella paramesenteroides*, *Klebsiella pneumoniae*, *Propionibacterium acnes J139*, *Clostridium xylanolyticum* were the dominant species that contributed to the differences in GH silages (Figure 5A and Figure S1). *Klebsiella pneumoniae*, *Weissella paramesenteroides*, *Pediococcus pentosaceus*, *Enterobacter hormaechei*, *Enterobacter hormaechei*, *Enterococcus faecium*, *Bacillus subtilis* were the mainly dominant species that contributed to the difference in SH silages (Figure 5B and Figure S2).

**Figure 6:**
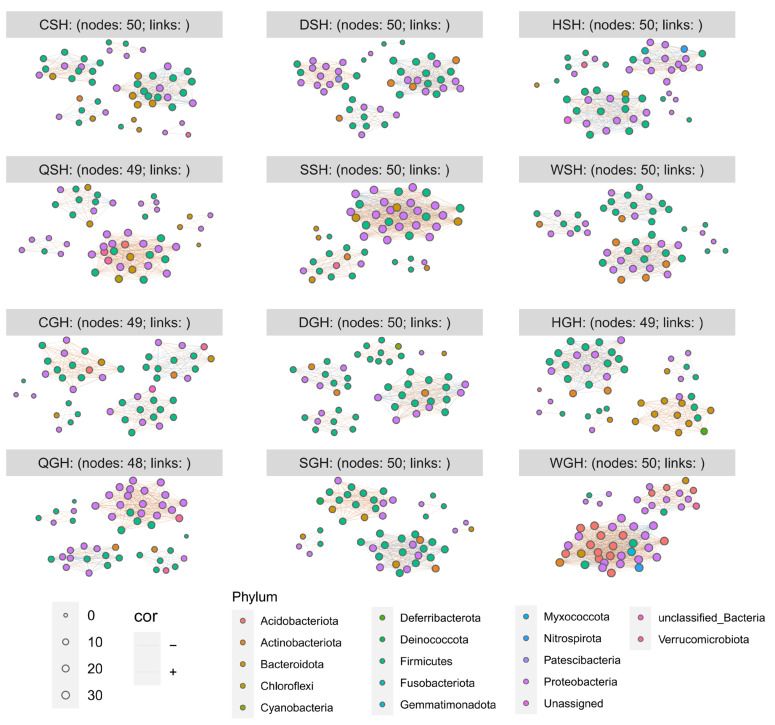
Bacterial co-occurrence networks of GH and SH silages with the most abundant 50 species (*p* value < 0.05, correlation > 0.6).

The networks of the bacterial community were analyzed using Spearman’s rank correlation to assess the influences of environmental factors on fermented natural forage grass (Figure 6). The highest edge number was found in the bacterial network of WGH (593), followed by SSH (514), while the lowest edge number was observed in CGH (285, Figure S3). The ranking of bacterial networks in terms of average degree in two kinds of grass was ranked as follows: WGH > SGH > QGH > HGH > DGH > CGH; SSH > DSH > QSH > HSH > WSH > CSH. All silages, except for the QSH and SSH groups, showed good relative modularity, suggesting highly modular bacterial communities, especially in the GH silage. The majority of edges in co-occurrence networks of bacterial community exhibited positive correlations.

### 3.4. The Correlation Between Environmental Factors and Bacterial Communities

**Figure 7:**
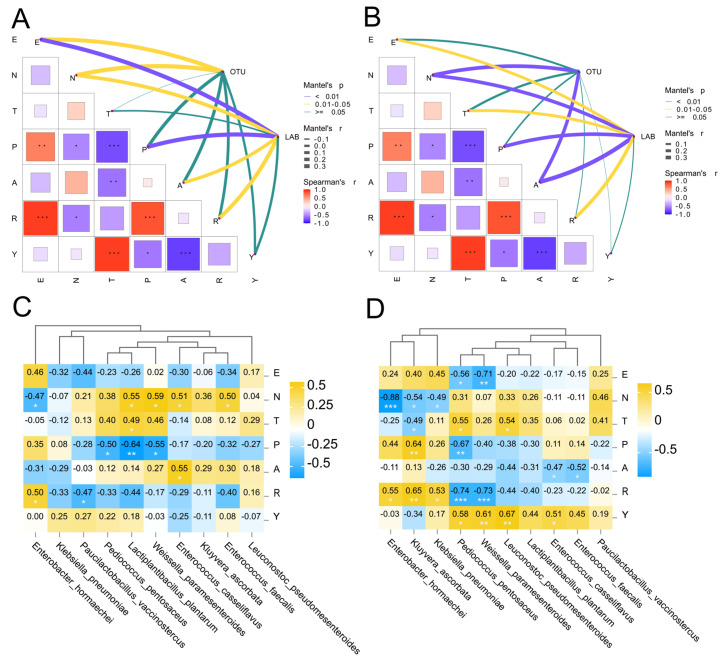
The Mantel test and Spearman correlation revealed relationships between the OTU, LAB and geographical factors ((**A**): GH; (**B**): SH). The Spearman’s correlation heatmap presents the relationships between the dominant bacterial species (Top 10) and geographical factors ((**C**): GH; (**D**): SH). *, *p* < 0.05; **, 0.001 < *p* < 0.01; ***, *p* < 0.001; E, longitude; N, latitude; T, temperature; P, precipitation; A, altitude; R, relative humidity; Y, sunshine duration.

To investigate the impact of environmental factors on bacterial communities of natural forage silage, the Mantel test was performed between the composition of forage silage microbiota and the environmental factors (Figure 7A,B). In GH silages, E (*p* < 0.05) and N (*p* < 0.05) were positively correlated with the OTUs. Besides Y and T, all environmental factors studied in this study had significant (*p* < 0.05) effects on LAB. N and A were important environmental factors influencing the bacterial OTUs in SH silages. In addition to Y, all other environmental factors studied in this study had significant effects on LAB, particularly altitude (r = 0.40, *p* < 0.01). Spearman’s correlation analysis was employed to further investigate the associations between dominant bacterial species (Top 10) and geographical environmental factors (Figure 7C,D). In GH silages, N demonstrated significant positive correlations with *Lactiplantibacillus plantarum* (r = 0.59), *Weissella paramesenteroides* (r = 0.55), *Enterococcus casseliflavus* (r = 0.51), and *Enterococcus faecalis* (r = 0.50), whereas a significant negative correlation was observed with *Enterobacter hormaechei* (r = −0.47). Additionally, P exhibited significant negative correlations with *Pediococcus pentosaceus* (r = −0.50), *Lactiplantibacillus plantarum* (r = −0.64), and *Weissella paramesenteroides* (r = −0.55). *Enterobacter hormaechei* exhibited a highly significant negative correlation with N (r = −0.88, *p* < 0.001), while *Pediococcus pentosaceus* and *Weissella paramesenteroides* showed highly significant (*p* < 0.001) negative correlations with R, with correlation coefficients of −0.74 and −0.73, respectively.

## 4. Discussion

### 4.1. Fermentation Quality

As shown in Figure 2A, *Paucibacter* and *Pantoea* were the most abundant bacteria in the two fresh materials. The genus *Paucibacter* has been detected in oat silage, but there have been few reports on their functional roles within silage [33]. *Pantoea* is a genus of Gram-negative bacteria frequently isolated from plant surfaces [10,34]. Studies have found a negative correlation between the genus *Pantoea* and NH_3_-N content, suggesting that this genus may have the ability to reduce NH_3_-N during ensiling [35], while others considered that the role of *Pantoea* in silage is similar to that of *Enterobacter*, as they compete with LAB for substrates [36,37]. Fortunately, in most terminal silage samples from this study, both genera exhibited relatively low abundance. The relative abundance of *Leuconostoc* in GH was 12.21%, while that in SH was only 0.04%. *Lactococcus* and *Leuconostoc* are dominant LAB commonly found in the early stage of silage fermentation [38,39]. Notably, *Leuconostoc* can rapidly initiate the fermentation process. The surface of fresh SH harbored a relatively low abundance of LAB, which may negatively impact its silage fermentation. *Pantoea ananatis* can secrete lignolytic enzymes and plays a significant role in the degradation of lignin in plant leaves [40,41]. But there have been no reports on its role in silage fermentation, which warrants further attention in the future. In this study, the high abundance of *Leuconostoc lactis* (44.07%) in S suggested that warm regions may promote the colonization of LAB on plant surfaces.

Silage pH is one of the most important indicators for assessing the quality of silage fermentation [35]. In this study, the pH values of all samples were higher. None of the samples met the standard for high-quality silage (pH < 4.2). Thus, the silages produced in this study should be classified as low-quality silage. *Saccharum arundinaceum* may contain relatively low level of WSC, which limits the substrate available for rapid LAB growth [27]. As for *Leucaena leucocephala*, its high crude protein content (which can be up to 18.95% DM) likely confers a high buffering capacity, which may resist pH decline and create a prolonged window for competitive microorganisms. Lactic acid is the primary organic acid responsible for lowering the pH value and inhibiting undesirable microorganisms during silage fermentation [13,42]. In this study, lactic acid content varied considerably by region. A high level of butyric acid, a product of *Clostridium* and *Enterobacter* fermentation, in silage is undesirable, as it not only leads to the loss of nutrients but its odor can also reduce the feed intake of animals [2,43]. Butyric acid was commonly present in GH silages, with the HGH group exhibiting an excessively high level. To sum up, it is necessary to apply LAB inoculants or chemical additives to improve the fermentation quality of silage in Hainan island. It is noteworthy that, in this study, the silage pH values of the two plants in D were all the highest, which may be due to the fact that the geographical environmental factors in D are not conducive to the fermentation of LAB.

### 4.2. Microbial Ecology

Alpha diversity is an important component of biodiversity and a comprehensive index reflecting richness and evenness. Based on the comprehensive analysis of ACE, Chao 1, Simpson, and Shannon indicators, this study revealed that geographical environmental factors exerted significant impacts on the bacterial abundance in GH silage, and showed greater influence on bacterial diversity in SH silage. Geographic locations exert a significant influence on the bacterial composition of silage.

As illustrated in Figure 4A, *Enterobacter* and *Klebsiella* were the dominant undesirable bacteria in GH silages which is an undesired observation. *Enterobacter* and *Klebsiella* are common undesirable microorganisms in high-pH silages [39,44]. These two genera, harboring abundant CAZyme genes and antibiotic resistance genes, always lead to dry matter loss and decrease the hygienic quality of silage [15,45,46]. In addition, studies by Zong et al. [47], have shown that even under a lower pH condition, the species of *Klebsiella* can survive by providing α-tocopherol to LAB. Therefore, it is necessary to control the abundance of these two genera during the silage fermentation process. A rich diversity of LAB, including *Weissella*, *Leuconostoc*, *Pediococcus*, and *Latilactobacillus*, was observed across different GH and SH silages, with their composition varying significantly depending on geographical origin and silage type. The relatively high LAB biodiversity observed in Hainan silages suggests that this tropical region might harbor distinct LAB strains. However, direct isolation, identification, and inoculation trials are required to confirm whether these strains possess superior silage-fermenting capabilities compared with known inoculants. *Weissella*, *Leuconostoc*, *Enterococcus*, and *Lactococcus* can rapidly initiate silage fermentation and are relatively active during the early stage of ensiling. However, they have poor acid tolerance, and as the pH of the silage decreases, they are often replaced by more acid-tolerant LAB, such as *Lactobacillus plantarum* [48,49,50]. The widespread dominance of these acid-sensitive LAB observed across silage samples in this investigation is likely attributable to substrate limitation, which restricted metabolic progression beyond initial fermentation stage.

According to Banerjee et al. [51], positive associations in bacterial co-occurrence networks reflect mutualistic or cooperative behavior between bacterial taxa. When the network exhibits high modularity and positive correlation dominance, cooperation within the network modules can buffer disturbances and thus result in higher stability [52,53]. In the context of silage, a highly modular bacterial community could confer stability by compartmentalizing functions: for instance, one module may drive rapid lactate production, while another module degrades residual plant polysaccharides without compromising pH decline. This functional redundancy within modules helps maintain fermentation homeostasis under perturbation. Generally, silage enters a stable phase after 60 days of ensiling (or even earlier in tropical regions), after which the bacterial community becomes relatively stable. In a stable bacterial community, the majority of microorganisms should exhibit mutualistic or cooperative relationships, which is consistent with the previous conclusions. In the bacterial co-occurrence networks of hybrid *Pennisetum* and Italian ryegrass silages during the stable phase, a predominance of positive edges over negative edges was also observed [54,55]. *Klebsiella*, recognized as a detrimental microorganism in silage, could survive by supplying α-tocopherol to *Lactobacillus plantarum* in fermented rice straw [47], confirming the previous conclusion that microorganisms in stable ecosystems are mostly mutualistic or cooperative. Higher complexity in WGH silage (593 edges) may reflect a less stabilized community with ongoing microbial turnover, which aligns with its relatively high pH (5.56). Future studies combining metatranscriptomics or metabolite profiling are needed to directly test the functional implications of network topology.

### 4.3. Environmental Drivers

In GH silages, E and N were positively correlated with the OTUs. This indicated that geographical location serves as a significant regional factor influencing bacterial communities in GH silages. Similarly, Zi et al. [22] have documented comparable findings in their investigation of bacterial communities associated with *Pennisetum sinese* silage across geographically distinct regions. Besides Y and T, all environmental factors studied in this study had significant effects on LAB. N and A were important environmental factors influencing the bacterial OTUs in SH silages. In addition to Y, all other environmental factors studied in this study had significant effects on LAB, particularly altitude. As previously mentioned, altitude was a critical geographical environmental determinant modulating LAB fermentation dynamics, with pronounced effects observed in high-altitude ecosystems. Excessive rainfall during silage production is undesirable. In tropical regions, the characteristic high precipitation levels coupled with pronounced seasonal variability significantly compromise silage production by disrupting optimal moisture management during both pre-ensiling field operations and anaerobic fermentation phases. The abundance of LAB exhibited a significant positive correlation with precipitation levels in the current study, a phenomenon potentially attributable to adequate rainfall sustaining continuous proliferation of LAB populations on fresh forage surfaces [19]. Furthermore, all geographical environmental factors in this study demonstrated measurable impacts on the silage fermentation microbiota of natural forage silage. Among these, latitude served as the shared geographical environmental factor influencing the silage bacterial community composition. This is consistent with the research results of Wu et al. [56]. The results implied that significant latitudinal variations may necessitate adjustments in processing methods for the same forage plant, such as modifying types of LAB inoculants or utilizing moisture-absorbing agents to optimize water content during silage preparation. Furthermore, the same bacterial species exhibited differential correlations with geographical factors across plant substrates, highlighting the necessity of tailored additive strategies based on specific raw materials and local environmental conditions during silage production.

### 4.4. Practical Implications

A high abundance of *Pediococcus* was observed in the DSH and CSH groups of SH silage (Figure 4), by which it was indicated that *Pediococcus* can dominate in SH silage. Therefore, *Pediococcus* could be considered a preferred microbial inoculant for SH silage production in the future. Similarly, *Weissella*, *Leuconostoc*, and *Lactiplantibacillus* were observed to dominate in GH silage, suggesting that a synthetic microbial consortium composed of these three genera could be considered an effective approach to improving the fermentation quality of GH silage. Furthermore, the fact that the dominant LAB species differed among silages indicates that it is necessary to use different inoculants for different forage species. On the other hand, environmental factor analysis identified latitude as the key driver of silage microbiota. In tropical regions, significant latitudinal variations may require adjustments in processing methods for the same forage plant, such as modifying LAB inoculants or using moisture-absorbing agents to optimize water content.

## 5. Conclusions

This study investigated the effects of geographical environmental factors on fermentation parameters and bacterial communities in *Saccharum arundinaceum* and *Leucaena leucocephala* silage. The results demonstrated significant variations in fermentation characteristics and bacterial community structures of the same plant species across different geographical regions. Geographical environmental factors including longitude, latitude, altitude, precipitation, and relative humidity were identified as influential factors shaping silage bacterial communities, with latitude being the common geographical factor affecting the bacterial communities in the two natural forage silages. Collectively, these findings highlight the critical need for geographical adaptation of production strategies, even when using the same plant, to account for local environmental conditions during practical silage manufacturing.

## Figures and Tables

**Table 1:** Fermentation parameters of natural forage silages in Hainan Island.

Items	Plants	Locations						Mean	*p* Value
		H	Q	W	S	C	D		
pH value	GH	5.25 ^c^	5.35 ^c^	5.56 ^b^	5.27 ^c^	5.04 ^d^	5.84 ^a^	5.39	**
	SH	4.32 ^d^	5.59 ^a^	5.32 ^b^	5.04 ^c^	5.26 ^b^	5.63 ^a^	5.19	**
Lactic acid (g/kg DM)	GH	37.20 ^a^	19.13 ^b^	40.65 ^a^	8.59 ^c^	18.55 ^b^	8.10 ^c^	22.04	**
	SH	70.67 ^e^	15.36 ^f^	170.40 ^b^	129.47 ^c^	89.65 ^d^	197.46 ^a^	112.17	**
Acetic acid (g/kg DM)	GH	7.68 ^b^	1.89 ^c^	12.52 ^a^	0.23 ^c^	7.67 ^b^	1.58 ^c^	5.26	**
	SH	7.09 ^b^	6.45 ^b^	18.54 ^a^	6.41 ^b^	6.45 ^b^	8.08 ^b^	8.84	**
Butyric acid (g/kg DM)	GH	16.21 ^a^	3.02 ^b^	2.65 ^b^	ND	2.80 ^b^	2.18 ^b^	5.37	**
	SH	ND	ND	ND	ND	ND	1.15	1.15	—

Notes: ND, not detected; H, Haikou; D, Danzhou; Q, Qiongzhong; W, Wangning; S, Sanya; C, Changjiang; Different lowercase letters in the same row indicate significant differences (*p* < 0.05); **, *p* < 0.01. The same below.

**Table 2:** Chemical composition of natural forage raw materials in Hainan Island.

Items	Plants	Locations						Mean	*p* Value
		H	Q	W	S	C	D		
DM %	GH	37.24 ^bc^	40.29 ^b^	33.46 ^c^	46.04 ^a^	38.69 ^b^	45.07 ^a^	40.13	**
	SH	33.07 ^b^	39.10 ^b^	39.25 ^b^	37.96 ^b^	35.92 ^b^	51.87 ^a^	39.53	**
CP % DM	GH	7.46 ^b^	7.01 ^b^	10.23 ^a^	10.02 ^a^	10.31 ^a^	9.83 ^a^	9.14	**
	SH	18.95 ^a^	11.38 ^c^	16.65 ^b^	10.03 ^c^	16.54 ^b^	11.82 ^c^	14.23	**
NDF % DM	GH	72.62 ^a^	74.82 ^a^	72.82 ^a^	72.14 ^a^	72.93 ^a^	73.03 ^a^	73.06	NS
	SH	51.28 ^ab^	43.03 ^ab^	41.21 ^b^	47.31 ^ab^	42.42 ^ab^	53.33 ^a^	46.43	**
ADF % DM	GH	40.67 ^a^	43.07 ^a^	40.16 ^a^	40.66 ^a^	40.80 ^a^	43.51 ^a^	41.48	NS
	SH	32.98 ^b^	42.03 ^a^	35.94 ^ab^	36.17 ^ab^	32.84 ^b^	31.26 ^b^	35.20	**

Notes: DM, dry matter; CP, crude protein; NDF, neutral detergent fiber; ADF, acid detergent fiber; NS, not significant.

## Data Availability

The raw sequence data have been deposited in the sequence read archive at the NCBI (https://www.ncbi.nlm.nih.gov/) under accession number PRJNA1265434.

## Supplementary Materials

The following supporting information can be downloaded at: https://www.mdpi.com/article/10.3390/microorganisms14040899/s1, Figure S1: LEfSe analysis histogram of LDA value distribution (GH silages); Figure S2: LEfSe analysis histogram of LDA value distribution (SH silages); Figure S3: Parameters of bacterial co-occurrence networks. A~D: Bar plots of GH silage bacterial co-occurrence network parameters (A: edge numbers; B: negative/positive correlation number; C: average degree; D: relative modularity). E~H: Bar plots of SH silage bacterial co-occurrence network parameters (E: edge numbers; F: negative/positive correlation number; G: average degree; H: relative modularity); Table S1: Geographical and environmental factors of sampling sites.
