# Supplementary material for: Geographic and Climatic Effects on Fermentation Quality and Bacterial Diversity of Saccharum arundinaceum and Leucaena leucocephala Silage
Source: Microorganisms. 2026 Apr 16;14(4):899. doi: 10.3390/microorganisms14040899 (PMC13118623; doi:10.3390/microorganisms14040899)
Supplement: Supplementary file 1 [file microorganisms-14-00899-s001.zip › microorganisms-4236842-supplementary.pdf]

Supplementary Materials

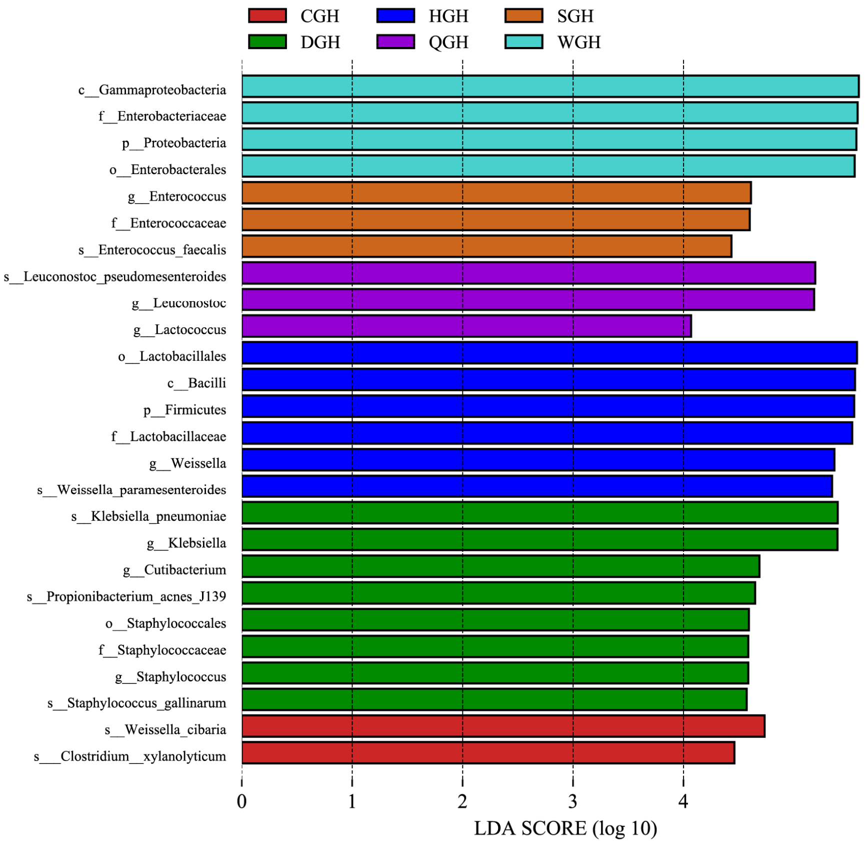

Figure S1 LEfSe analysis histogram of LDA value distribution (GH silages).

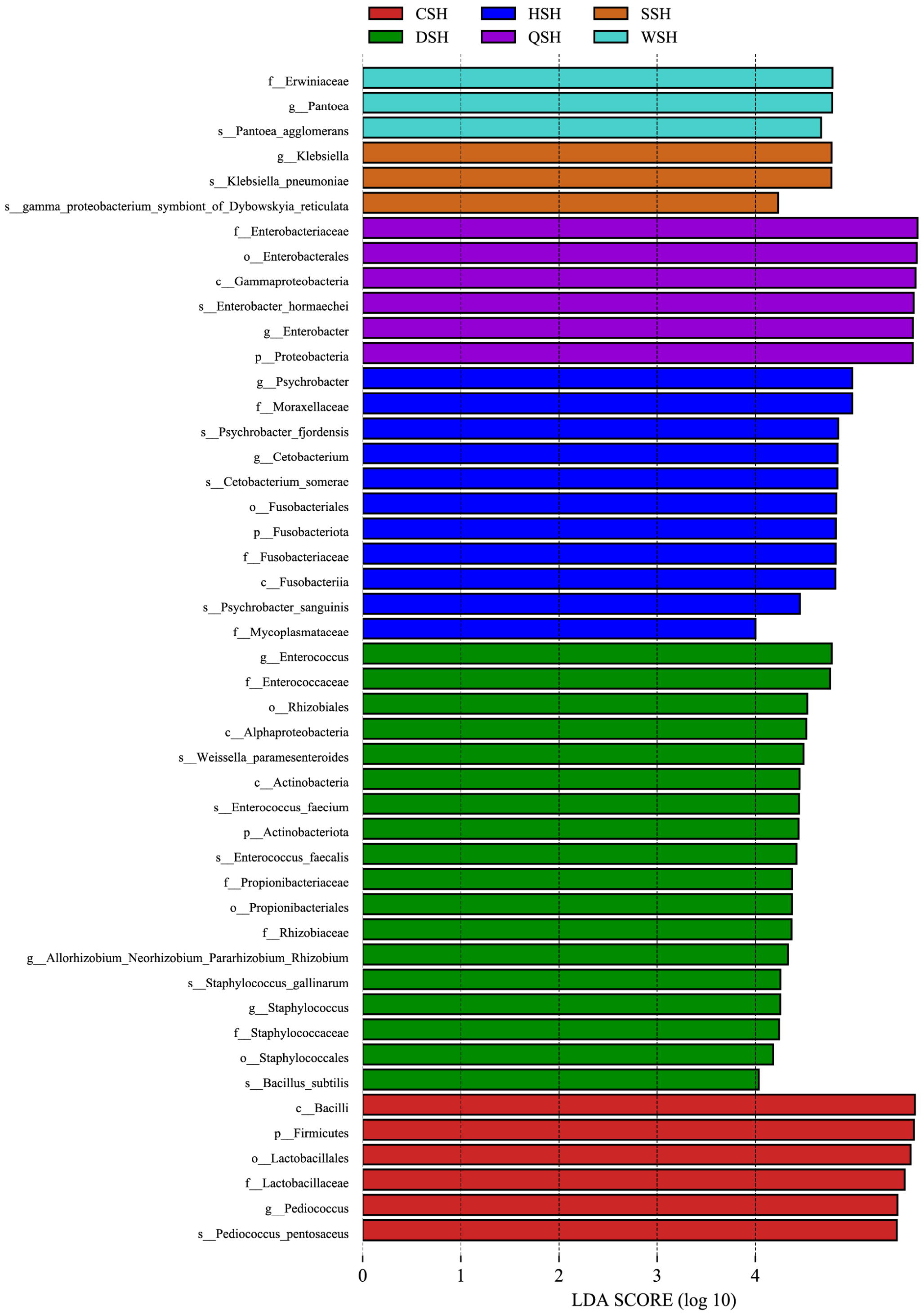

Figure S2 LEfSe analysis histogram of LDA value distribution (SH silages).

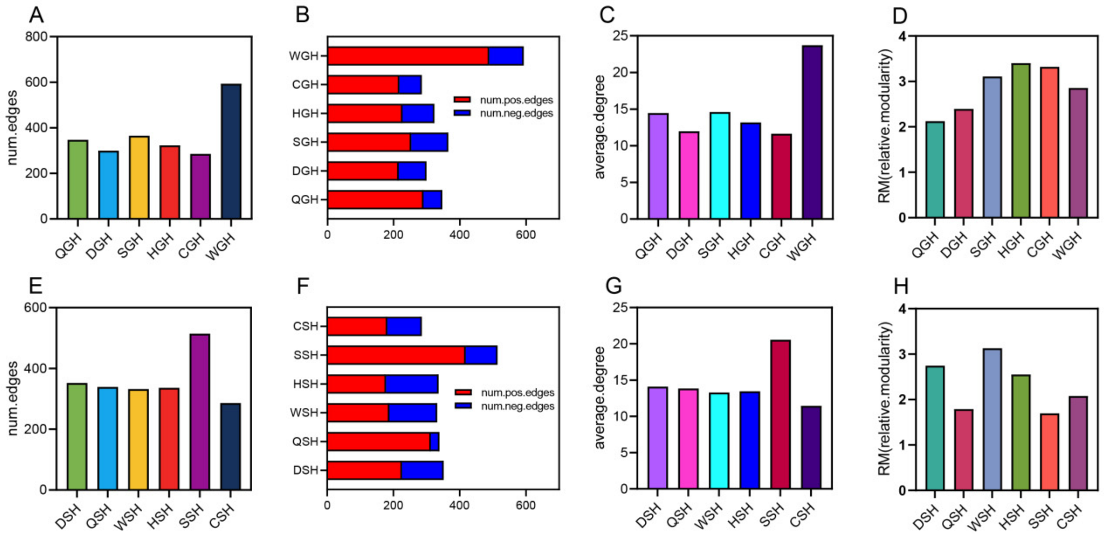

Figure S3 Parameters of bacterial co-occurrence networks. A~D: Bar plots of GH silage bacterial co-occurrence network parameters (A: edge numbers; B: negative/positive correlation number; C: average degree; D: relative modularity). E~H: Bar plots of SH silage bacterial co-occurrence network parameters (E: edge numbers; F: negative/positive correlation number; G: average degree; H: relative modularity).

Table S1 Geographical and environmental factors of sampling sites

Locations	E(°)	N(°)	T（℃）	P（mm）	A(m)	H(%)	S(h)
Changjiang	108.97	19.32	24.6	1680	90	73.57	2081.5
Danzhou	109.50	19.50	23.8	1870	160	76.99	1857.5
Haikou	110.18	19.87	24.1	1788	160	80.82	1856.7
Qiongzhong	109.57	19.02	22.4	2350	560	81.44	1843.6
Sanya	109.57	19.32	26.9	1716	40	77.27	2168.7
Wanning	110.42	18.90	23.9	2420	30	82.85	1965.5

Notes: E, longitude; N, latitude; T, temperature; P, precipitation; A, altitude; R, relative humidity; Y, sunshine duration
